# Prevalence of Foreign Body Aspiration in Children in a Tertiary Care Hospital

**DOI:** 10.31729/jnma.5393

**Published:** 2021-02-28

**Authors:** Kripa Dongol, Yogesh Neupane, Heempali Das Dutta, Bigyan Raj Gyawali, Bijaya Kharel

**Affiliations:** 1 Department of ENT, Tribhuvan University Teaching Hospital, Maharajgunj, Kathmandu, Nepal

**Keywords:** *airway*, *aspiration*, *children*, *choking*, *foreign body*

## Abstract

**Introduction:**

Foreign body aspiration is a common problem in children with significant mortality and morbidity. This study aims to determine the prevalence of foreign body aspiration in children in a tertiary care hospital of Nepal.

**Methods:**

A descriptive cross-sectional study was conducted at Tribhuvan University Teaching Hospital from April 2010 to March 2016 after obtaining ethical approval from Institutional Review Committee (Reference number- 08(6-11)E277/78). All children of age up to 15 years with suspected foreign body aspiration were included. The data was collected from the medical record section and entered in Microsoft Excel. The descriptive statistical analysis was performed.

**Results:**

A total of 26,294 patients were included in the study. The prevalence of foreign body aspiration in children was found to be 98 (0.37%). On rigid bronchoscopy, 82 patients (83.6%) were confirmed to have a foreign body in the airway. The peak incidence of foreign body aspiration was seen in patients of age group one to two years. The commonest foreign body in the airway was a peanut.

**Conclusions:**

The prevalence of foreign body aspiration in children was low, which is similar to other studies. Foreign body aspiration may lead to dreadful complications. Therefore, both the clinicians and the public need to be cautious about it.

## INTRODUCTION

Foreign body aspiration could be a life-threatening situation. It is estimated to cause 350 to 2000 deaths in the United States annually.^1^ Usually, male children of age less than three years are at high risk because of immature dentition, poor pharyngeal reflex, and a tendency to explore surroundings by mouth.^2,3^ Nuts and seeds are commonly aspirated materials.^4^ The clinical manifestations are often nonspecific, which could lead to delay in diagnosis or in referral.^5^ Every effort should be made to prevent aspiration or early diagnosis and treatment to reduce the complications.^6^

Foreign body aspiration is a common cause for visiting the emergency department. However, there are no studies in Nepal that emphasize the importance of this problem and the clinicians should be aware of it.

This study's aim was to determine the prevalence of foreign body aspiration in children in a tertiary care hospital in Nepal.

## METHODS

This was a descriptive cross-sectional study conducted in the department of ENT, Tribhuvan University Teaching Hospital, Kathmandu, Nepal, from April 2010 to March 2016. Ethical approval for the study was obtained from the Institutional Review Committee (Reference number-08(6-11)E277/78). The whole sample technique was used in this study. Record files of children of all gender with age up to 15 years with suspected foreign body aspiration were assessed retrospectively. The minimum sample size was estimated using the following formula:


$$\begin{array}{ll} \text{n} & ={\text{Z}}^{\text{2}}\times \text{p}\times \text{q}/{\text{e}}^{\text{2}}\\ & ={\left(2.576\right)}^{2}\times 0.5\times (1-0.5)/{\left(0.007\right)}^{\text{2}}\\ & =26000 \end{array}$$

Where,

Z = 2.576 for confidence interval at 99%
p = prevalence 50%
q = 1-p
e = margin of error 0.7%

The medical records of the patients enrolled in the study were reviewed. The data collection included demographic information, symptoms and signs of foreign body aspiration, duration, site and type of foreign body aspiration, radiological findings and complications of aspiration and/or procedure. Presentation within 24 hours of aspiration was classified as an early presentation and presentation late than 24 hours was classified as a late presentation in this study. We used Microsoft Excel for data recording and analysis.

Rigid bronchoscopy was performed immediately for patients with severe signs and symptoms of respiratory distress with falling saturation. In contrast, for stable patients, the procedure was preferably performed in the daytime as an elective procedure. Patients were kept in the pediatric intensive care unit after the procedure. An experienced ENT surgeon performed rigid bronchoscopy. If rigid bronchoscopy failed in the first attempt, it was repeated after 48 hours by a more experienced surgeon. If the foreign body could not be removed after second attempt, it was removed by the cardiothoracic team via open thoracotomy.

## RESULTS

**Figure 1.**
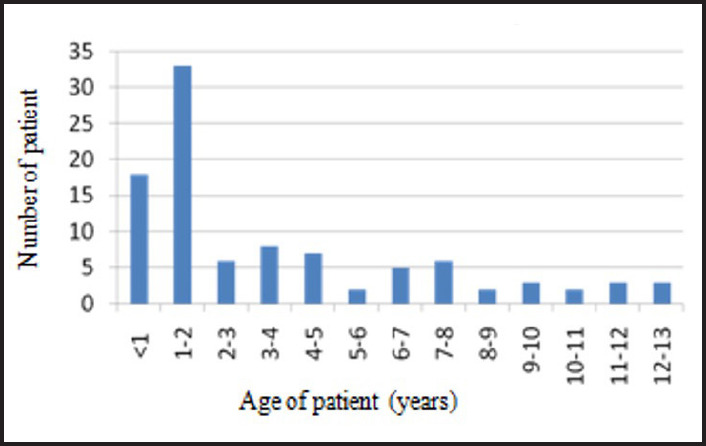
Number of patients in different age groups (n = 98).

There was a total of 26,294 children visiting the ENT department during six years of study, and 98 patients were admitted for suspected foreign body aspiration. Therefore, the prevalence of foreign body aspiration in children during the study period was calculated to be 98 (0.37%). There were 67 (68.3%) boys and 31 (31.7%) girls, with male to female ratio of 2:1. The patients' mean age was 4 ± 3.55 years, with an age range from six months to 13 years. The peak incidence of foreign body aspiration was seen in patients of age group 1-2 years. The age distribution of patients with foreign body aspiration is shown in figure 1.

Out of 98 patients with suspected foreign body bronchus, the foreign body was found in 82 (83.6%) patients, 14 (14.2%) patients didn't have a foreign body in their airway and 2 (2.04%) patients died prior to the procedure.

There were various types of foreign body aspirated, as shown in table 1. The commonest foreign body was a vegetative material found in 46 (56%) patients, with peanuts and seeds being frequently aspirated. Most of the foreign bodies lodged in the right bronchial tree i.e., 40 (48.8%). The site of lodgement of a foreign body in the airway is shown in table 2.

**Table 1:** Table showing different types of foreign body aspiration (n = 82).

Types of foreign body	Number of cases n (%)
Vegetative	46 (56)
Peanut	15 (18.3)
Seed	11 (13.4)
Soyabean	6 (7.3)
Bean	4 (4.9)
Maize	3 (3.7)
Rice	2 (2.4)
Coconut	2 (2.4)
Leaf	2 (2.4)
Pea	1 (1.2)
Non vegetative	36 (44)
Plastic whistle	10 (12.2)
Nail, pin	7 (8.6)
Plastic pen cover	6 (7.3)
Stone	2 (2.4)
Metallic objects	2 (2.4)
Bone	2 (2.4)
Charger tip	1 (1.2)
Chocolate	1 (1.2)
Bullet	1 (1.2)
Pen tip	1 (1.2)
Screw bolt	1 (1.2)
Aluminum foil	1 (1.2)
Metallic earring	1 (1.2)

**Table 2:** Table showing sites of foreign body lodgement (n = 82).

Site of foreign body lodgement	Number of cases n (%)
Right bronchial tree	40 (48.8)
Left bronchial tree	36 (44)
Subglottis	3 (3.6)
Trachea	2 (2.4)
Supraglottis	1 (1.2)

Fifteen patients (15.3%) presented early, i.e., within 24 hours of foreign body aspiration, whereas 83 (84.7%) of the patients presented late. The shortest duration of presentation to the hospital following foreign body aspiration was six hours and the longest duration was one year. Among the late presenters, nine patients were initially treated as pneumonia in another center. The most common presentations were cough and difficulty in breathing. The common examination findings were unilateral decreased breath sounds and wheeze. Only 51 (52%) of patients had a definite history of foreign body aspiration. The clinical features of foreign body aspiration are shown in table 3.

**Table 3:** Table showing clinical features of foreign body aspiration (n = 98).

Symptoms / signs	n (%)
Cough	77 (78.5)
Difficulty in breathing	46 (47)
Noisy breathing/stridor	30 (30.6)
Cyanosis	10 (10)
Fever	14 (14.2)
Unilateral decreased breath sounds	53 (54)
Normal breath sounds	21 (21.4)
Wheeze	19 (19.3)
Crepts	9 (9.1)

Chest X-ray was performed in all 98 patients. Fifty-three (54.1%) patients had normal chest X-rays. Radiopaque foreign body was seen in 24 (24.5%) patients. Atelectasis was seen in 11 (11.2%), consolidation in 7 (7.14%), hyperinflated lungs in 2 (2.04%) and hilar opacification in 1 (1.02%) patient.

The foreign body was failed to be retrieved during rigid bronchoscopy in the first attempt in 6 (6.2%) patients. All the six patients were late presenters. Three of the six were two years old and the other three were three, six, and ten years old, respectively. The foreign bodies in these cases were nuts (three cases), a plastic pen cover (two cases) and a nail (one case). In four patients, it was removed during the second attempt of rigid bronchoscopy and two patients underwent thoracotomy after the failed second attempt.

Out of 98 patients, complications during rigid bronchoscopy were seen in 5 (5.2%) patients. One patient developed pneumothorax who was managed with chest tube insertion and later recovered. Four patients had severe pre and intraoperative hypoxia, two of them had a good recovery, but unfortunately, the other two died.

There were 6 (6.12%) mortalities due to foreign body aspiration. Two patients died in the emergency even before rigid bronchoscopy could be performed. They were six months and 14 months of age, presented within hours of foreign body aspiration and had unilateral lung collapse on the chest X-ray. The other four patients died during or after rigid bronchoscopy due to hypoxia. Among these four patients, three patients were below one year of age and one was five years old.

## DISCUSSION

Foreign body aspiration is an underestimated problem. It is a preventable cause of morbidity and mortality in the pediatric population. In this study, the prevalence of foreign body aspiration in children was found to be 0.37% which is similar to the study done by Tseng HJ, et al.^7^ There was a high incidence of foreign body aspiration in male children below two years of age. Studies by Wolach et al.^2^ Salih et al.^3^ and Sink et al.^5^ have also stated a high incidence of foreign body aspiration in male children below three years of age. This may be because of incomplete dentition, the immature neuromuscular reflex of the upper aerodigestive tract, the tendency of toddlers to explore any objects in their vicinity and lack of constant supervision by the caretaker.^4,6^ Male children may be more prone to foreign body aspiration because of their adventurous and impulsive behavior.^8^

Organic foreign bodies are the ones that are commonly aspirated, with peanuts being the commonest.^4^ However, the type of foreign body could vary according to culture and food habits.^3^ In this study, peanuts and seeds were common vegetative foreign bodies. Whistle, nail and pin and rear cover of the plastic pen were common non-vegetative foreign bodies. Caretakers should be precautious about the possibility of choking these objects. While feeding the children, the nuts, legumes and grains should be fed in small pieces and they should always be supervised while feeding. Sidell et al. have reported the round organic foreign body to be the commonest cause for fatal asphyxia.^4^

Nearly 50% of the foreign bodies were found in the right bronchial tree and 44% in this study's left bronchial tree. This finding is similar to the studies done by Sidell et al.^4^ and Rizk and Rassi.^9^ Foreign body in the right main bronchus is common in adults because it is wider, shorter and vertical than the left main bronchus.^3^

In children, these anatomical differences between the right and left bronchi are less prominent and therefore, they have an equal distribution of foreign bodies.^10^ Yang et al. found a higher incidence of foreign bodies in the left bronchus in their study.^11^ Usually, the foreign body which impacts into supraglottis, tracheal lumen, carina and the mainstem bronchi could prove fatal.^4^

Only 15.3% of the patients presented early after suspected foreign body aspiration in our study. A large number of patients presented late, with the longest duration being one year after suspected aspiration. The delayed presentation of patients could be due to lack of knowledge of parents about worse consequences of aspiration, negligence of caretakers, public unawareness, a misdiagnosis by the clinicians and lack of facilities where rigid bronchoscopy is done. Naragund et al. found only 13.6% of patients presenting within 24 hours of aspiration.^12^ Chen and Zhang found 46.4% of patients presenting within 24 hours and 53.6% of patients presenting late. They concluded that delayed presentation leads to a significant increase in complications, prolonged operation time and hospitalization duration.^13^ Chance of granulation tissue formation, pus formation and risk of bleeding increases with duration of aspiration. The prolonged retention of foreign body in the airway leads to pneumonia, emphysema, atelectasis and bronchiectasis.^11^ Hygroscopic nature of organic foreign body leads to progression from partial to complete airway obstruction with prolonged duration of aspiration.^10^

The most common symptoms in this study were cough and difficulty in breathing. Sink et al. have reported cough, choking, and wheezing to be the common clinical presentations. History of cough had 88% sensitivity and only 18% specificity to predict foreign body aspiration. They have reported choking events may not be a good predictor of foreign body presence or absence.^5^ However, Sidell et al. have reported that 93% of the studies included in his review article have correlated a witnessed choking event with confirmed airway foreign body.^4^ In our study, 52% of patients had a definite history of aspiration. The lack of definite history of aspiration in our study could be due to the ambulatory nature of children so that they may have been out of parental sight during aspiration. Sometimes, parents may forget or neglect the history of aspiration, especially during a late presentation.

Unilateral decreased breath sounds and wheezing were common clinical signs in our study. The sensitivity and specificity of unilaterally decreased breath sound range from 53% to 80% and from 42.3% to 88%, respectively.^5^ The sensitivity of wheezing was found to be 58% and the specificity was found to be 64% by Sink et al.^5^

Most of the foreign bodies were radiolucent. Only 24.5% were radiopaque on chest X-ray in our study. This is similar to the studies done by Sidell et al.^4^ and Sink et al.^5^. In our study, 54.1% of patients had normal chest X-rays. Eren et al. also found two-thirds of their patients having normal chest radiography.^14^ A normal chest X-ray does not rule out the foreign body in the airway as there may not be any finding in the first 24 hours.^15^ The common findings in chest X-ray are air-trapping/hyperinflation, atelectasis, consolidation, and pulmonary infiltration.^4,7^ In some cases, computed tomography (CT) of the chest may be required if history, physical examination, and chest X-ray findings are confusing. Fluoroscopic dynamic evaluation of bilateral diaphragms has been advocated to detect unilateral bronchial foreign bodies.^7^ Flexible bronchoscopy is popularly being used nowadays in cases where there is doubt about foreign bodies in the airway.

In this study, 6.2% of patients had to undergo repeated rigid bronchoscopy. After failed second rigid bronchoscopy, 2.1% of the patients underwent open thoracotomy for foreign body removal. Williams et al. have stated the necessity of repeated bronchoscopy in 1 to 2% of the patients and thoracotomy in 0 to 6% of the patients.^6^ Rigid bronchoscopy is technically a highly demanding procedure requiring a well-experienced surgeon, anesthesiologist and pediatric critical care team. The difficulties during the procedure are encountered mostly in smaller children due to narrow airway diameter, especially if a large foreign body gets impacted in the subglottis. The other causes of difficulties may be due to spherical shape of the foreign body, airway edema, bronchospasm, prolonged duration of foreign body impaction, granulation tissue formation, increased secretion and bleeding.^4,5,10^

In our study, the complication rate of rigid bronchoscopy was 5.2%, with hypoxia being the commonest one. Kaur et al. have reported the complication rate to be 10%.^10^ The common complications are laryngeal edema, bronchospasm, pneumothorax, pneumomediastinum, cardiac arrest, tracheal or bronchial laceration and hypoxic brain injury.^5^ The mortality rate in this study due to foreign body aspiration was 6.12% which is higher than that reported by Kaur et al., which was 2%.^10^ In our study, 2% of patients died in an emergency even before rigid bronchoscopy could be performed, which suggests that aspiration could often be a dire emergency and we do not get time to plan the approach. All the five patients who died were below 18 months of age except one five-year-old child. Foreign body aspiration could prove lethal in smaller children either due to choking or due to the procedure.

Foreign body aspiration is a significant yet underacknowledged and underrepresented public health issue in the pediatric population.^4^ There are no national guidelines for the processing and packaging of food items or toys in our country. Therefore, safety guidelines should be formulated by the stakeholders with their strong implications in practice. Legislations enforcing mandatory labeling of the toys and food items about choking hazards should be developed.^6^ The public, especially the parents, caretakers, and teachers, should be educated and aware of the dangers of foreign body choking and its delayed treatment. Children should always be supervised while feeding and playing. Any sort of physical or emotional activity should be prohibited while feeding a child. Clinicians should be suspicious about foreign body aspiration in cases with recurrent chest infections and early referral of such cases to the otorhinolaryngologist is necessary.

The limitations of this study were its retrospective design, less sample size and a single-institution study.

## CONCLUSIONS

The prevalence of foreign body aspiration in children was low, which is similar to other studies. Foreign body aspiration is common in male children of age one to two years. Peanuts and seeds are commonly aspirated foreign bodies. Foreign body aspiration is a preventable life-threatening condition. Early diagnosis and treatment is essential to overcome its hazards.
